# Deterioration of Concrete Under the Combined Action of Sulfate Attack and Freeze–Thaw Cycles: A Review

**DOI:** 10.3390/ma18184309

**Published:** 2025-09-14

**Authors:** Hairong Wu, Chenjie Lv, Youliang Xu, Yuzhou Sun, Songzhao Qu, Xiangming Zhou

**Affiliations:** 1 School of Civil and Transportation Engineering, Henan University of Urban Construction, Pingdingshan 467036, China; lcj022418110@163.com (C.L.); sunyz@zut.edu.cn (Y.S.); qusongzhao@huuc.edu.cn (S.Q.); 2 China Railway Bridge Bureau Group Co., Ltd., Wuhan 430050, China; ztdqjzlb@163.com; 3 Department of Civil and Environmental Engineering, Brunel University London, London UB8 3PH, UK; xiangming.zhou@brunel.ac.uk

**Keywords:** concrete, freeze–thaw cycle, sulfate attack, damage mechanism, damage model, numerical simulation

## Abstract

The synergistic interaction between freeze–thaw cycles and sulfate attack induces a more severe and complex deterioration mechanism in concrete than either factor in isolation. This review elucidates this process by first examining the individual damage mechanisms and then integrating current research to analyze the coupled effects, revealing a complex process involving the superposition and competition of physical crystallization, chemical reactions, and fatigue stresses. The deterioration is delineated into four distinct stages: (1) Pre-Inflection Acceleration, (2) Post-Inflection Acceleration, (3) Deceleration, and (4) Rapid Failure. Experimental methodologies, research materials, and study protocols are critically examined, with particular emphasis on the influence of sulfate solution type and concentration, while highlighting significant discrepancies between laboratory conditions and field exposure. Based on this, the existing durability damage models and multi-physics numerical simulation methods are summarized, emphasizing the importance of cross-scale studies. Finally, prioritized research directions are proposed, emphasizing the need for refined experimental protocols and integrated physico-chemical models to advance predictive durability assessment. This work provides a foundational reference for guiding future research in concrete durability.

## 1. Introduction

**Figure 1:**
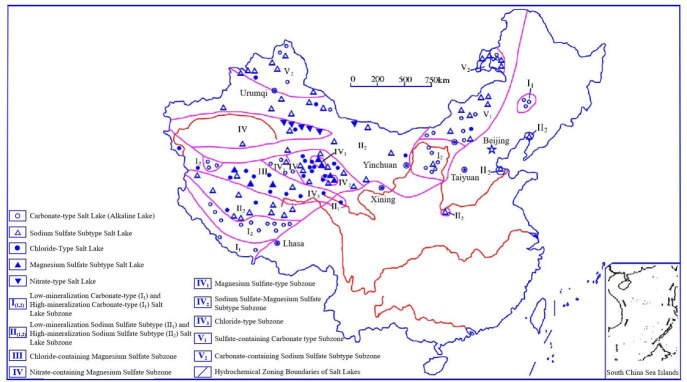
Distribution of salt lakes in China according to types of chemical compositions [4,9].

Concrete is one of the most widely used construction materials worldwide. However, due to inherent limitations in concrete itself and the complex variability of external environments, concrete structures suffer from durability deterioration, leading to reduced service life of buildings and Portland cement concrete (PCC) pavements [1,2]. In China, saline–alkali land is prevalent [3], where soil and water contain high concentrations of corrosive sulfate and chloride ions. Particularly, the saline soils in Northwest China are rich in sulfate ions (Figure 1) [4], and this region is also located in severe cold zones. Concrete structures serving in cold saline areas are subjected to multiple synergistic factors. When concrete structures are subjected to freeze–thaw cycles (FTCs) and sulfate attack (SA), sulfate ion penetration leads to both physical and chemical erosion, resulting in crystallization pressure and expansive products [5]; additionally, under negative temperature conditions, the internal solution freezes, generating hydrostatic pressure [6] and osmotic pressure [7]. The mechanism of durability degradation in concrete under combined FTC and SA is significantly more complex than those under individual SA or FTCs [8], making concrete structures in such environments particularly vulnerable to potential durability issues.

Research on deterioration mechanisms of concrete under freeze–thaw cycles (FTCs) [10,11,12] and sulfate attack (SA) [13], along with associated predictive models [14] and numerical simulations [15,16,17], has achieved considerable maturity. Recent scientific focus has shifted toward damage mechanisms and quantitative evaluation of concrete subjected to combined sulfate attack and freeze–thaw cycling. Jiang et al. [18,19] reported that in the early stages of the experiment, concrete in 5% Na_2_SO_4_ solution exhibited lower compressive strength loss and reduced relative dynamic modulus of elasticity (RDEM) loss compared to water-immersed specimens. For instance, after 400 freeze–thaw cycles, the compressive strength loss of concrete in 5% Na_2_SO_4_ solution was 39.6%, compared to 37.7% in water. While mass loss in sulfate solution was consistently reported to be lower than in water throughout testing, damage progression accelerated markedly during later stages. Notably, deterioration severity in 1% Na_2_SO_4_ exceeded that observed in both 5% Na_2_SO_4_ and pure water environments.

Contradicting trends emerged in Hu et al.’s study [20], where water exposure caused more severe damage before 100 FTCs, whereas 5% Na_2_SO_4_ specimens demonstrated maximal deterioration after 200 cycles. Conversely, Liu et al. [21] documented through ultrasonic testing that 3.5% NaCl, 5% sulfate, and composite solutions all mitigated FTC damage during short-term exposure (200 rapid FTCs ≈ 2 months). This protective effect was attributed to insufficient duration for sulfate corrosion development, freezing-point depression by sodium sulfate, and enhanced compressibility of ice in saline solutions.

Zhu et al. [22] employed nuclear magnetic resonance (NMR) to quantify pore structure evolution in manufactured aggregate concrete under SA-FTC coupling. Their analysis revealed that sulfate solutions can reduce freeze–thaw deterioration rates, with the reduction effect generally becoming more significant as sulfate concentration increases. Low temperatures suppressed chemical sulfate attack, shifting dominance toward physical damage mechanisms. Elevated sulfate concentrations further depressed freezing points, resulting in slower deterioration rates compared to pure water exposure.

Although substantial research exists on damage evolution under SA-FTC coupling—examining reaction products, macro-/micro-properties, and structure–property relationships—methodological inconsistencies in mix designs, curing regimes, and testing protocols have prevented consensus formation. Li et al. [23] established a Hydraulic-Thermal-Salt-Mechanical (HTSM) coupled model to simulate salt-frost heaving in U-shaped concrete channels on saline soils under FTCs. The model captures moisture–salt migration, phase change, and Na_2_SO_4_ crystallization, and is validated through outdoor experiments. Overall, there is limited research on the quantitative evolution of macroscopic and microscopic damage in concrete under sulfate–freeze–thaw conditions [24].

This comprehensive review synthesizes current understanding across three critical domains: deterioration mechanisms, damage modeling, and numerical simulation approaches for concrete durability under SA-FTC coupling. Based on critical analysis, prioritized research directions are proposed to address existing knowledge gaps.

## 2. Deterioration Mechanisms of Concrete Under Combined Action of Sulfate Attack and Freeze–Thaw Cycles

Concrete constitutes a multiphase heterogeneous material comprising solid phases (aggregate, calcium silicate hydrate (C-S-H), portlandite (CH), AFm/AFt phases, and unhydrated cement particles), liquid phases (partially saturated pore water, capillary water, adsorbed water, interlayer water, and chemically bound water), and gaseous phases (entrapped air voids and microcrack-contained gases). Its microstructure exhibits inherent heterogeneity and structural complexity. Its microstructure exhibits inherent heterogeneity and structural complexity. The pore structure of concrete, particularly microcracks and micropores, provides transport pathways for gases, water, saline solutions, and other deleterious media. Besides mix proportion parameters, microstructural characteristics—including the type, quantity, and spatial distribution of solid phases and pores—exert governing control over the engineering characteristics of hardened concrete: strength, dimensional stability, and durability [5].

### 2.1. Deterioration Mechanism of Concrete Under Freeze–Thaw Cycles

Freeze–thaw deterioration in concrete originates from cyclic fatigue damage caused by repeated frost-induced expansive pressures and osmotic pressures generated during phase changes in free pore water under temperature fluctuations. This process manifests as surface scaling and degraded mechanical properties [1]. The pore structure fundamentally governs frost resistance, with key classifications including: gel pores (0.03–3 μm; non-damaging), capillary pores (1–50 μm; primary damage sites), and air voids—accidental (100–5000 μm from mixing/vibration) versus entrained (5–25 μm via air-entraining agents) [25].

Since the 1850s, four principal frameworks have emerged—Hydrostatic Pressure Theory [6], Osmotic Pressure Theory [7], Critical Water Saturation Theory [26], and Micro-Ice Lens Theory [27]—with the Hydrostatic Pressure Theory and the Osmotic Pressure Theory being most widely validated.

**Figure 2:**
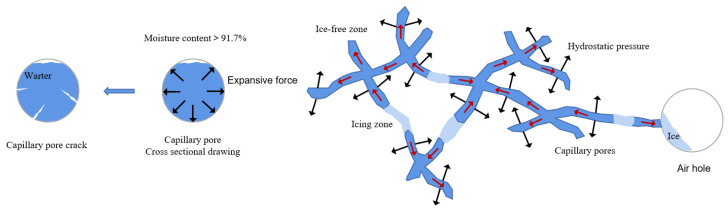
Hydrostatic pressure generation diagram (Red arrow in capillary pores: flow direction).

According to the Hydrostatic Pressure Theory [6], the capillary pores constitute primary damage zones, while gel pores (water freezing point < −78 °C) [5] and air voids (incompletely saturated, typically either dry or partially filled with water) remain minimally affected [5]. The sequential mechanism (Figure 2) initiates when environmental water infiltrates capillary pores. Subsequent temperature drops lead to the crystallization of freezable water in capillary pores, expanding volume by 9% and forcing unfrozen water migration from the frozen areas to the outside, generating a hydrostatic pressure. If the water is expelled into adjacent air voids (e.g., the pores connected to capillary pores, as shown on the right side of Figure 2), these air voids provide effective escape space, reducing and releasing the hydrostatic pressure. When the water migration path is sufficiently short, the resulting hydrostatic pressure does not exceed the ultimate tensile strength of the cement paste, thus causing no damage to the concrete. However, if no air voids are present nearby, the hydrostatic pressure cannot be relieved. As the water content in the capillary pores increases and exceeds 91.7%, the pore walls experience significant expansive forces. This generates high tensile stresses around the capillary pores. Once these stresses surpass the internal ultimate tensile strength of the concrete, the capillary pores crack, leading to structural damage. Pressure relief occurs when water drains into adjacent air voids (Figure 2). Damage initiates under three cumulative conditions: (1) water migration paths exceed critical length, (2) capillary saturation exceeds 91.7%, and (3) resultant pressures surpass the cement paste’s tensile strength. Essentially, the Hydrostatic Pressure Theory attributes damage to spatially constrained ice crystallization, exerting radial stresses on pore walls.

**Figure 3:**
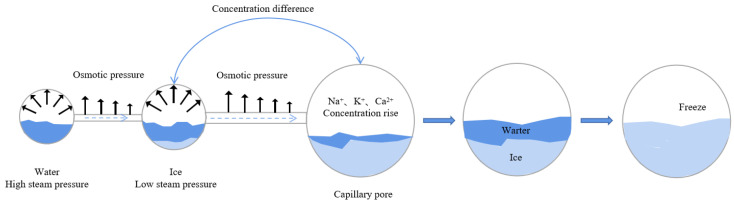
Schematic diagram of osmotic pressure production.

The Osmotic Pressure Theory [7] explains freeze–thaw damage scenarios lacking significant volumetric expansion. When capillary pore solutions contain ions (e.g., Na^+^, K^+^, Ca^2+^), the mechanism progresses as follows (Figure 3): First, pore solution freezing point depression occurs inversely with pore diameter due to surface tension effects. Larger capillaries freeze first, concentrating solutes in remaining liquid and establishing chemical potential gradients between larger (frozen) and smaller (unfrozen) pores. This concentration differential drives unfrozen solution migration from smaller to larger capillaries, generating concentration-induced osmotic pressure. Concurrently, the lower vapor pressure of ice versus water at identical temperatures creates vapor pressure gradients, forcing additional solution flow toward frozen zones and producing vapor-induced osmotic pressure. When the combined osmotic pressure exceeds the concrete’s tensile strength, microcracking initiates and propagates.

Both Hydrostatic Pressure Theory and Osmotic Pressure Theory elucidate freeze–thaw damage through fluid migration mechanisms in pore systems (capillary/air voids) following ice formation. These frameworks focus on interactions between ice, unfrozen solution, and vapor pressure, where complex physical transformations generate destructive pressures that mechanically degrade concrete. Complementarily, the Critical Water Saturation Theory [26] posits that damage severity correlates directly with internal moisture content—higher saturation intensifies frost-induced expansive forces. Meanwhile, the Micro-Ice Lens Theory [27] proposes that freeze–thaw cycling accentuates water absorption from the environment, progressively elevating saturation and exacerbating damage with cumulative cycles.

Despite the fundamental importance of microstructure–property models for understanding concrete performance, their practical engineering applicability remains limited due to concrete’s inherent heterogeneity, dynamic behavior, and microstructural complexity [5]. To bridge this gap, our research group [10] established a novel damage scaling model based on the Hydrostatic Pressure Theory, using environmental cooling rates as the key parameter. This approach facilitates the utilization of extensive laboratory accelerated freezing test data for predicting frost durability lifespan in real-world exposure conditions.

### 2.2. Deterioration Mechanism of Concrete Under Sulfate Attack

**Figure 4:**
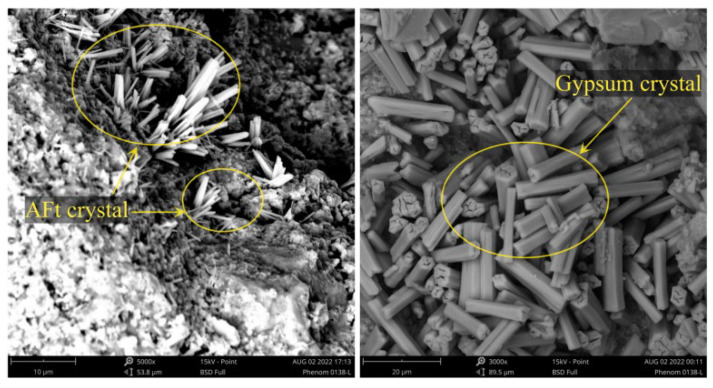
Expansion products AFt and gypsum under sulfate freeze–thaw cycle [30].

Sulfate attack on concrete represents a crystallization-driven deterioration process categorized into physical and chemical mechanisms [28]. Following SO_4_^2−^ ingress, physical sulfate attack manifests through salt crystallization in pores: anhydrous Na_2_SO_4_ transforms into mirabilite (Na_2_SO_4_·10H_2_O) with 315% volumetric expansion [29]. Concurrent chemical attack involves sulfate reacting with hydration products, forming expansive gypsum (CaSO_4_·2H_2_O) and ettringite (3CaO·Al_2_O_3_·3CaSO_4_·32H_2_O) (Figure 4) [30,31], thereby inducing internal stresses and microcracking [32]. This complex physicochemical degradation [31] exhibits mechanism variations depending on sulfate species and concentration. Al-Amoudi [33,34] classification delineates three distinct modes: (1) acidic attack (pH < 11.4), where C-S-H decalcification generates gypsum, converting concrete into cohesionless granular material with reduced cross-section and strength [35]; (2) expansive attack in alkaline media, characterized by ettringite formation from SO_4_^2−^-Ca(OH)_2_-C_3_A reactions causing expansion/cracking; and (3) spalling attack in mixed sulfates, exhibiting surface delamination through flaky or shell-like scaling.

**Figure 5:**
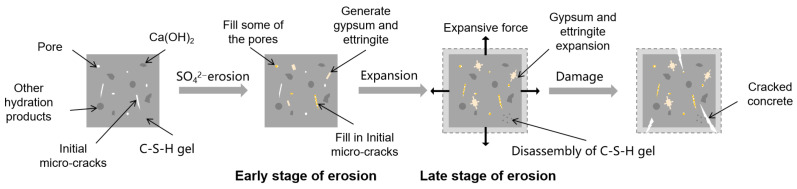
Schematic diagram of concrete damage by sulfate attack.

The multifaceted nature of sulfate attack mechanisms [36] results in coexisting deterioration pathways and reaction products across concrete depths [37]. Critical immersion conditions dictate damage modes: partially immersed mortar undergoes combined physical salt crystallization and chemical sulfate attack due to capillary action, whereas fully immersed specimens experience solely chemical degradation via diffusion-controlled ion transport [38]. Fundamentally, sulfate-induced damage stems from synergistic interactions between crystallization pressures (physical) and expansive reaction products (chemical). As depicted in Figure 5, this degradation evolves through sequential phases: Initial reaction products partially occupy pores and microcracks, enhancing concrete density and mass during Phase I. Subsequently, in Phase II, accumulated products exceed pore accommodation capacity, generating expansive stresses that induce internal cracking, surface spalling, and structural disintegration [39]. Concurrent chemical transformations of hydration products—particularly the structural transition of C-S-H from cohesive to friable states [31]—synergistically accelerate microcrack initiation and propagation throughout the deterioration process.

### 2.3. Deterioration Mechanism of Concrete Under Coupled Sulfate Attack and Freeze–Thaw Cycles

**Figure 6:**
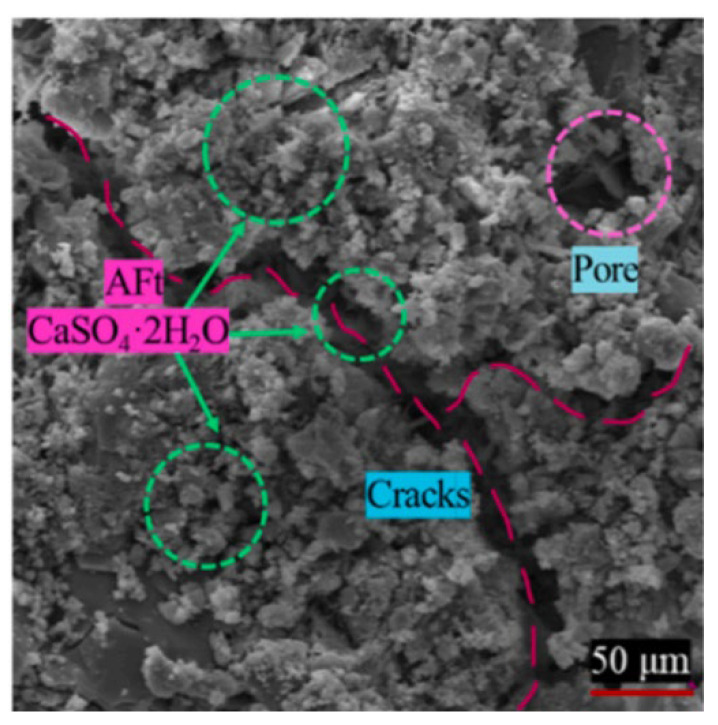
Microscopic image of concrete after 200 freeze–thaw cycles [42].

**Figure 7:**
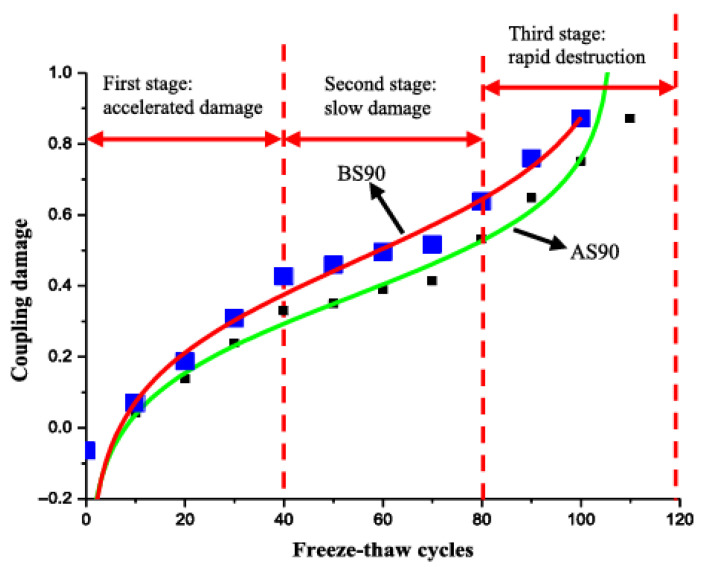
Three stages of concrete damage under coupled sulfate attack and freeze–thaw cycles [44] (AS represents w/c of 0.45, BS represents w/c of 0.55, 90 represents the number of freeze–thaw cycles, and sulfate concentration is 20%; coupling damage refers to the synergistic effect of SA-FTCs).

Under coupled freeze–thaw and sulfate attack conditions, concrete undergoes a multifaceted deterioration process characterized by interacting damage mechanisms. The combined and interactive effects of salt solution and freeze–thaw cycles exhibit dual mechanisms [40]. (1) Freezing-point depression mitigates FTC damage, while (2) FTC-induced salt crystallization generates destructive pressures [41] that accelerate expansion cracking. Under combined exposure, low temperatures suppress sulfate reaction kinetics [19], yet increased initial saturation from sulfate solutions elevates hydraulic pressures within capillary pores during freezing. Critically, late-stage sulfate ingress produces expansive corrosion products (ettringite/gypsum) that hydrate and swell. When superimposed with FTC-induced stresses, these combined forces exceed the concrete’s tensile strength, initiating expansive cracking (Figure 6 [42]) [43]. Both References [44,45] classify the damage progression of concrete under combined sulfate attack and freeze–thaw cycles into three sequential phases (Figure 7): The Accelerated Phase demonstrates temporary mitigation through sulfate pore-filling and freezing-point depression; the Decelerated Phase reflects competing mechanism equilibrium; and the Rapid Failure Phase exhibits synergistic FTC-SA deterioration [45]. Xiao et al. [46,47] confirmed analogous phase-progressive damage in recycled aggregate concrete under identical exposure conditions, validating this failure progression framework.

**Figure 8:**
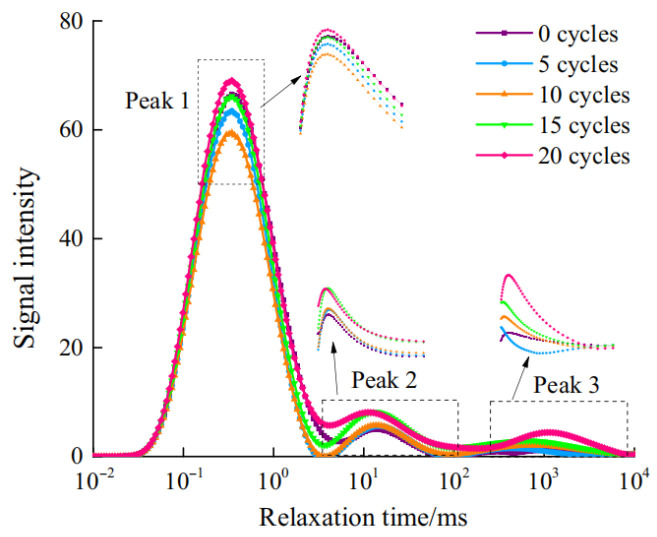
T2 spectrum distribution curve (T2, transverse relaxation time from NMR analysis).

**Figure 9:**
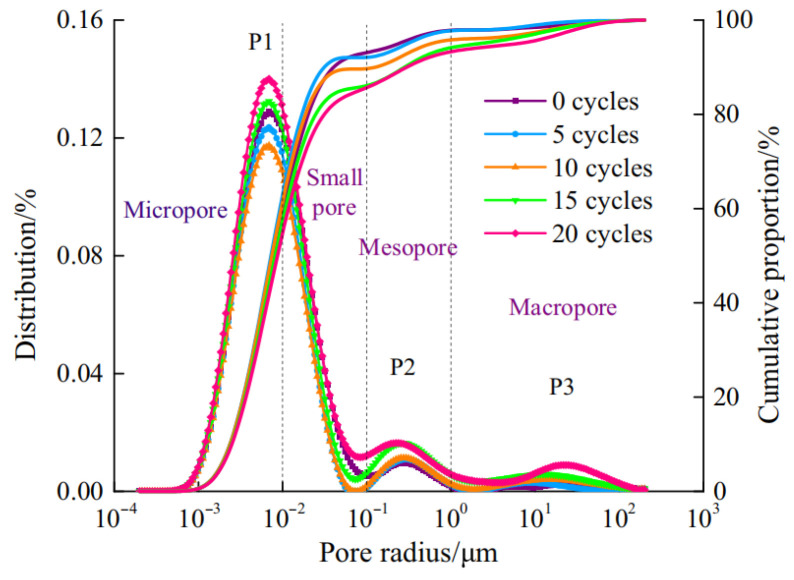
Division of pore radius.

Under the combined action of SA and FTCs, experimental investigations [48] (specimens: w/c = 0.58, 28-day curing, 150 d hydrostatic immersion, 3 d natural drying; cyclic exposure: 20% Na_2_SO_4_ immersion for 10 h → −20 °C freezing for 8 h → 65 °C drying for 6 h, to simulate real-world durability conditions) revealed progressive pore structure evolution: After 5 cycles (considered the initial phase), original pores became filled with ettringite and gypsum from sulfate–cement reactions, increasing density and impermeability (Figure 8). By 10 cycles (considered the intermediate phase), mesopore (2–50 nm) and macropore (>50 nm) proliferation emerged due to frost-induced crack propagation and widening. At 15 cycles (advanced phase), continued enlargement of meso/macropores reduced fractal dimension (Figure 9), indicating structural simplification. After 20 cycles (considered the terminal phase), accumulated ettringite/gypsum exceeded pore accommodation capacity, generating expansive pressures that enlarged pores and created preferential pathways for salt ingress. This accelerated frost damage propagation, ultimately increasing total porosity through synergistic degradation mechanisms.

**Figure 10:**
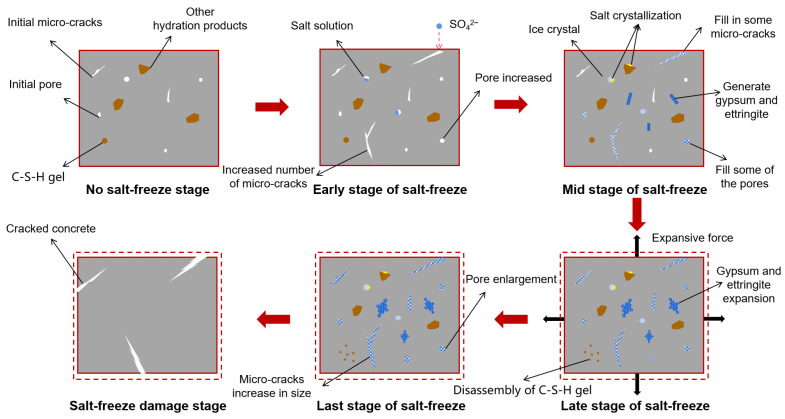
Schematic diagram of concrete sulfate freeze–thaw failure process.

Consensus exists regarding late-stage synergistic acceleration of damage beyond individual exposures; however, early-stage interactions—including sulfate-induced freezing-point depression, reaction product formation kinetics, and cryogenic suppression of sulfate attack—remain inadequately resolved. Building upon seminal triphasic damage models under sulfate freeze–thaw coupling [44,45] and leveraging microstructural insights into pore-stress–permeability relationships [45], this study synthesizes current mechanistic understanding [43,49,50,51,52] to propose a refined four-phase deterioration framework (Figure 10).

Stage I (the initial stage of salt freezing, Pre-Inflection Accelerated Phase): Capillary-driven water/SO_4_^2−^ ingress facilitates FTC-dominated damage initiation [53], increasing surface microcrack density that subsequently enhances ionic penetration pathways [49].

Stage II (the early stage of salt frost, Post-Inflection Accelerated Phase): Pore solution crystallization initiates ettringite/gypsum precipitation [42] that refines pore structure—reducing capillary/macropore volume [48]—thus improving impermeability and temporarily impeding ion transport [54]. Concurrent freezing-point depression [40] and cryogenic reaction retardation promote pore-filling dominance over expansion [48], collectively suppressing deterioration rates.

Stage III (the later stage of salt frost damage, Decelerated Phase): Expanding reaction products transition from pore-filling to expansive mechanisms [48]. FTC-induced hydraulic pressures co-occur with sulfate-driven C-S-H disintegration [39], while increased meso-/macropore connectivity elevates saturation gradients [51]. Counterbalancing effects emerge: sulfate-enhanced ice compressibility [55] and microcrack coalescence partially offset frost damage [45], maintaining near-Stage II deterioration rates with gradual acceleration.

Stage IV (the final stage of salt frost damage, Rapid Failure Phase): Reaction products exceed pore accommodation capacity, exerting expansive stresses [48]. Concurrently, concentrated solutions generate crystallization pressures [45], synergizing with FTC-induced hydraulic forces and sulfate expansion to produce triaxial tensile stresses [52]. This triggers cascading pore-coarsening, crack propagation, and mechanical property degradation [18].

The FTC-SA deterioration framework presented in this study derives primarily from controlled laboratory investigations. Under actual field exposure, damage mechanisms exhibit significantly heightened complexity due to dynamic interdependencies among environmental variables: cation speciation (e.g., Na^+^ vs. Mg^2+^), sulfate concentration gradients, ambient pH fluctuations [56], freezing rate, minimum freezing temperature, cryogenic exposure duration, and cyclic saturation states [10] collectively induce non-linear interactions absent in accelerated testing. These site-specific conditions fundamentally alter reaction kinetics, ice nucleation behavior, and damage progression pathways—rendering field deterioration mechanisms substantially more complex than laboratory simulations can capture.

## 3. Damage Model of Concrete Durability Under Combined Action of Sulfate Attack and Freeze–Thaw Cycles

As discussed above, the synergistic damage mechanism between SA and FTCs exhibits particular complexity, with mutual inhibition or acceleration effects occurring at different stages of interaction. The factors influencing the rate of SA, listed in order of decreasing importance, are as follows [34]: cement type, sulfate type and concentration, concrete quality, and exposure conditions. Currently, researchers primarily focus on the material level of concrete, using key control variables such as salt solution concentration and type, water–cement ratio, admixtures, and supplementary cementitious materials to conduct freeze–thaw resistance tests in sulfate solutions. Based on experimental results, durability damage and service life prediction models for concrete under combined SA and FTCs have been established. These models enable quantitative analysis of concrete performance and service life degradation under dual environmental actions.

### 3.1. Experimental Research

Table 1 summarizes the experimental studies conducted by domestic scholars on freeze–thaw tests using various types and concentrations of salt solutions. It provides detailed test parameters, including: initial exposure age of specimens, specimen dimensions, water–binder ratio (with special notations for cases involving manufactured sand vs. aeolian sand, fiber reinforcement, recycled aggregate concrete, air entrainment, silica fume, etc.). For subsequent discussions regarding the durability damage model of concrete under SA-FTCs coupling action, the specific experimental conditions can be referenced directly from Table 1 without repetitive elaboration in the text.

As summarized in Table 1, most studies employ rapid freezing tests per Chinese standard GB/T 50082 [73] or American standard ASTM C666 [74] to evaluate concrete salt-frost resistance. In these tests, sulfate solutions replace water in specimen containers during standardized freeze–thaw cycling. Alternative approaches utilize specialized environmental chambers [52,58] to simulate combined SA-FTC exposure. Recent work increasingly aims to develop regionally customized testing regimes [63,66], tailoring freeze–thaw duration to local climatic conditions to better replicate in-service environmental exposure and enhance durability assessment accuracy.

Nevertheless, current findings remain largely limited to laboratory investigations of specific mixtures under controlled freeze–thaw protocols. Significant knowledge gaps persist regarding systematic analysis of thermal regime parameters (e.g., cooling rates, minimum freezing temperatures, dwell times) and field validation of laboratory-derived models for engineering applications.

### 3.2. Erosion Models

#### 3.2.1. Durability Damage Models Based on Macro-Test Indicators

Models of concrete freeze–thaw damage are predominantly developed through freeze–thaw cycling (FTC) experiments. These experiments correlate the number of FTCs with key macro-mechanical properties, including mass loss rate, dynamic elastic modulus, ultrasonic pulse velocity, and compressive strength. Subsequently, regression analysis is employed to derive performance evolution models [75]. Adopting this methodology, many researchers have investigated the combined effects of salt attack (SA) and FTCs. Their experimental findings form the basis for establishing durability damage models utilizing these macro-scale indicators. Experimental parameters for the models discussed herein are detailed in Table 1.

Lu et al. [52] designed a concrete durability test investigating the combined effects of corrosive salts and FTCs in Northwest China, establishing correlations between mass loss, compressive strength, and service life. They developed a Birnbaum–Saunders distribution-based prediction model for concrete durability life, demonstrating its efficacy in characterizing damage evolution under salt-FTC synergy.

Dong et al. [42,69] evaluated capillary water absorption in aeolian sand concrete under SA-FTC coupling, analyzing the impacts of (i) aggressive environments (5% Na_2_SO_4_, 10% Na_2_SO_4_, 5% Na_2_SO_4_ + 3.5% NaCl) and (ii) aeolian sand content (0%, 20%, 100%). They identified strong correlations between freeze–thaw damage degree and both initial absorption rate and capillary penetration depth. Using Wiener stochastic-process-based deterioration indices, they predicted concrete residual life, determining compressive strength loss rate as the most sensitive indicator.

Zhang et al. [67] conducted durability tests on early-age concrete (w/b = 0.26, 0.32, 0.38) in Ruoqiang, Xinjiang, under three exposures: FTCs alone, SA alone, and SA-FTC coupling. A variable-weight buffered GM(1,1) model integrated with gray system theory and genetic algorithm (GA) predicted service life reductions under SA-FTC versus FTCs alone: 75.3→30.7 years (w/b = 0.26), 24.6→22 years (w/b = 0.32), and 15.3→12.7 years (w/b = 0.38). Subsequently [68], they quantified entropy-weighted durability values from morphology, mass loss, strength, and dynamic modulus data (air-entraining agent: 0.00%, 0.05%, 0.10%, and 0.15%), proposing a GM-GA-BP neural network model for durability prediction.

Duan et al. [64] established a damage attenuation model for fiber-reinforced concrete under: (i) composite salt–freeze/thaw cycles and (ii) composite salt–dry/wet cycles. The model employs ultrasonic pulse velocity damage parameter as the independent variable and relative residual splitting tensile strength as the dependent variable, achieving correlation coefficients > 0.95, confirming its validity in characterizing strength degradation.

Jiang et al. [76] extended Cai Hao’s FTCs-only elastic modulus evolution model [77], experimentally deriving the degradation behavior of mechanical properties under SA-FTC coexistence. They proposed an elastic modulus damage evolution equation (Equation (1)) for this combined exposure.

(1) $$5\%{\mathrm{Na}}_{2}{\mathrm{SO}}_{4}\;\mathrm{Solution}:\;\bar{E}=E_{0}(1-0.00275N{)}^{0.6379}\;5\%{\mathrm{MgSO}}_{4}\;\mathrm{Solution}:\;\bar{E}=E_{0}(1-0.00253N{)}^{0.93365}$$

where $E_{0}$ and $\bar{E}$ are the elastic modulus of concrete before and after damage, respectively; *N* is the number of FTCs.

Different from standard indoor testing conditions, Wang et al. [66] developed an accelerated laboratory erosion protocol to simulate the combined effects of wet–dry cycles, corrosive ions, extreme temperatures, and freeze–thaw cycles (FTCs) prevalent in Western China’s saline soils. This protocol—designed to evaluate sulfate erosion resistance of ultra-high-performance concrete (UHPC) and enable laboratory assessment of real-world durability—comprised four phases per 40-day cycle: high-temperature exposure, ambient-temperature exposure, FTCs, and low-temperature exposure. Each cycle represents ~5 years of field exposure using a 10% Na_2_SO_4_ solution (5× field concentration). Using compressive strength as a degradation metric, they established a Wiener stochastic process-based service life prediction model for UHPC.

Nevertheless, durability damage models relying on macro-test indicators face inherent limitations: (i) prohibitive duration of coupled SA-FTC tests, (ii) inadequate replication of real environmental complexity, (iii) excessive experimental resource demands, and (iv) limited data output. Consequently, such models cannot comprehensively characterize concrete freeze–thaw deterioration mechanisms.

#### 3.2.2. Models Based on Damage Accumulation and Probability Distribution

Aiming to predict concrete service life, Guan et al. [78] conceptualized durability failure under multifactorial exposure as a process of progressive internal damage accumulation. They established a universal multivariate Weibull distribution model based on reliability theory and continuum damage mechanics. Specific to SA-FTC deterioration, they derived a generalized damage evolution equation (Equation (2)) [79], validated through durability experiments on concrete with water-cement ratios of 0.28, 0.32, 0.36, and 0.40. Specimens were subjected to both standard rapid freeze–thaw cycles (ASTM C666) and salt–freeze–thaw exposure in 5% ammonium sulfate solution. Results demonstrated the model’s robust applicability under freeze–thaw conditions.

(2) $$E(D)=N^{-2}\sum_{i=0}^{\frac{N}{2}-1}4(N-2i-1)\left\{1-\exp\left\{-{\left[\lambda_{i}{\left(0.001m-k_{0}\lambda_{i}^{-1}\right)}_{+}\right]}^{\alpha })\right\}\right\}$$

where *D* represents the degree of damage; *N* is the number of divisions per side (even number); *I* = 0, 1, 2 …… (*N*/2 − 1); *m* is the freeze–thaw cycle number related to the time scale; *k*_0_ is the undetermined proportionality constant; *α* and $\lambda_{i}$ are the shape factor and scale factor of the Weibull distribution, respectively; and the subscript “+” means that if the value in parentheses is negative, it is set to 0, otherwise unchanged.

A growing body of research introduces damage variables derived from experimental data to quantify concrete degradation states. Scholars conceptualize crack-containing concrete as a damage-field-defined continuum, framing performance degradation as the evolution of this damage field to establish performance evolution equations. Duan et al. [64] developed a damage degradation model to characterize the deterioration of fiber-reinforced concrete under combined salt–freeze–thaw/cyclic wetting–drying conditions. Based on the theory of material decay, the model employs ultrasonic pulse velocity damage as the independent variable and relative splitting tensile strength as the dependent variable (Equation (3)), effectively characterizing the deterioration of fiber-reinforced concrete under combined salt–freeze–thaw and wet–dry cycles with high correlation (R^2^ > 0.95).

(3) $$R=\frac{1}{1+aD_{v}^{b}}$$

where *R* denotes the relative splitting tensile strength, and *D*ᵥ represents the ultrasonic pulse velocity damage, calculated as *D*ᵥ = (*v*_0_ − *v*ₙ) / *v*_0_, in which *v*ₙ is the pulse velocity after *n* cycles and *v*_0_ is the initial pulse velocity. The fitting parameters *a* and *b* were determined using MATLAB.

Xie et al. [65] investigated the multi-scale damage characteristics of fiber-reinforced aeolian sand concrete under salt–freeze coupling. By leveraging the Weibull distribution to quantify the probability distribution of micro-damage elements, they derived a micro-damage variable and established a damage constitutive model for concrete under salt erosion and freeze–thaw conditions. The bonding properties at cement-based interfaces were characterized using normal and tangential spring elements from the Linear Parallel Bond Model (LPBM). Through secondary development of the LPBM source code, they introduced a particle contact damage factor at ball–ball interfaces, constructing a discrete-random damage particle flow model for fiber-reinforced aeolian sand concrete.

Xiao et al. [46,47] studied the physico-mechanical properties of recycled aggregate concrete (RAC) with replacement ratios of 0%, 30%, 50%, and 100% under combined SA-FTC exposure. Based on microstructural and reaction product analyses, they elucidated the composite damage mechanism of RAC and established a Weibull-distributed damage equation using two-parameter Weibull statistics.

(4) $$D_{n}=1-\exp\left[-{\left(\frac{n}{\mu }\right)}^{\beta }\right]$$

where *D*_n_ represents the damage variable, *n* denotes the number of freeze–thaw cycles, *μ* is the proportion factor, and *β* is the Weibull shape factor. For ordinary concrete, *μ* is 3878.347 and *β* is 0.819.

Wei et al. [30] examined the deterioration patterns in both macro- and micro-properties of recycled aggregate concrete (RAC) subjected to combined SA and FTCs. Macroscopic indicators—including surface damage, mass loss rate, and relative dynamic modulus of elasticity (RDEM)—were assessed for RAC with varying recycled coarse aggregate (RCA) replacement ratios (0%, 50%, 100%) and moisture contents (0%, 50%, 100%). Utilizing nuclear magnetic resonance (NMR) and microhardness testing, the evolution of multiple micro-parameters was investigated: porosity, pore size distribution, and the width and strength of the interfacial transition zone (ITZ). A key finding was that the fractal dimension of RAC decreased under erosion, supporting its utility as a critical index for characterizing pore distribution complexity. Furthermore, a model describing pore structure damage during FTCs was established by integrating pore parameters η (accounting for both structural integrity and distribution specificity), and is expressed as follows:

(5) $$D_{\eta }=1-\frac{\eta_{n}}{\eta_{0}}$$

where $D_{\eta }$ is the modified micro-damage variable, $\eta_{n}$ is the integrated pore parameter of damaged concrete, $\eta_{0}$ is the initial integrated pore parameter of concrete, and *n* is the number of freeze–thaw cycles.

Wang et al. [44] investigated the durability of concrete in saline soil environments under the combined effects of carbonate–sulfate compound erosion and freeze–thaw cycles. They conducted three test regimes: carbonate–sulfate erosion, FTCs, and coupled salt erosion–freeze–thaw tests, establishing a predictive model for concrete damage in saline soil regions (Equation (6)):

(6) $$D=1+\frac{E_{n}}{E_{0}}\left[\alpha -\beta \ln\left(\frac{k}{N}-\gamma \right)-1\right]$$

where *D* is the damage variable, *N* is the number of freeze–thaw cycles, *E*_0_ and *E*_n_ are the initial elastic modulus and elastic modulus of concrete after *N* cycles, and *a*, *β*, *γ*, *k* are the undetermined parameters calibrated by experiments.

Qiu et al. [80] investigated the performance degradation of marine concrete structures exposed to seawater freeze–thaw cycles (FTCs) in cold regions. By analyzing stress–strain relationships and damage variables after 0, 25, 50, 75, 100, and 125 seawater FTCs, the authors developed a constitutive model (Equation (7)) based on plastic damage theory and experimental data to characterize concrete behavior under seawater FTCs.

(7) $$D_{c}=1-\frac{\sigma_{c}E_{c}^{-1}}{\varepsilon_{c}^{pl}\left(\frac{1}{b_{c}}-1\right)+\sigma_{c}E_{c}^{-1}}$$

where *D*_c_ is the damage to concrete under uniaxial compression, and *E*_c_ is the initial stiffness and of the specimen under uniaxial compression, *σ*_c_ is the loading stress of the specimen under uniaxial compression, $\varepsilon_{c}^{pl}$ is the plastic strain of concrete under compression, *b*_c_ is the proportion of plastic strain in concrete under compression relative to inelastic strain, with a recommended value in the range of 0.7–0.9.

Zhu et al. [70] developed a method for quantifying SO_4_^2−^ concentration in hardened concrete. Through numerical analysis, the relationship between sulfate diffusion depth and freeze–thaw cycles (FTCs) was investigated, revealing time-dependent diffusion behavior in manufactured sand concrete under varying FTC counts. Their findings identified 100 FTCs as a critical threshold under combined sulfate attack (SA) and FTC exposure. Below this threshold, high sulfate concentrations primarily drive crystallization-induced expansion and accelerated diffusion. Beyond 100 FTCs, freezing of sulfate solutions inhibits ion diffusion, leading to progressively reduced diffusion depths in high-concentration environments. Accounting for Northwest China’s harsh conditions, the authors established a sulfate diffusion model for manufactured sand concrete under FTCs:

(8) $$\frac{X}{X_{0}}={\left(1-\frac{N}{N_{0}}\right)}^{\upsilon }e^{\upsilon \frac{N}{N_{0}}}$$

where *N* is the number of FTC, *X* is the diffusion depth of SO_4_^2−^ at N times of FTC, *X*_0_ is the diffusion depth of SO_4_^2−^ at the failure of the sample, and *v* is the parameter used to characterize the diffusion.

The inherently heterogeneous internal composition of concrete, characterized by its multi-scale micro-/meso-structures spanning from nano-scale cement hydrates to millimeter-scale aggregates, critically governs its macroscopic properties. Furthermore, coupled environmental physical fields—including stress, temperature, and seepage fields within concrete structures—significantly modulate freeze–thaw damage characteristics [75]. Current research demonstrates that the reliability of damage models is directly dictated by the selection and definition of damage variables; however, these models often fail to elucidate the intrinsic multi-physics mechanisms governing the multifaceted factors affecting concrete frost resistance. Therefore, grounded in freeze–thaw damage theory, it is imperative to establish a comprehensive, mechanistically rigorous frost resistance model through theoretical derivation, integrating the interactions of multiple factors, and to implement high-fidelity numerical simulations of durability damage. This integrated approach is paramount for ensuring the safety and long-term serviceability of infrastructure in cold regions.

## 4. Numerical Simulation of Concrete Damage Under Combined Sulfate Attack and Freeze–Thaw Cycles

Significant progress has been made in numerical simulation of concrete durability damage under isolated freeze–thaw cycles (FTCs) or sulfate attack (SA). For single-factor FTCs, researchers primarily employ two methodological approaches. The first utilizes microscale cement paste models based on hydro-thermo-mechanical (HTM) tri-field coupling to analyze ice crystallization pressure [81]. The second uses mesoscale frameworks that incorporate aggregates, cement paste, and the interfacial transition zone (ITZ), which are simulated via discrete element method (DEM), finite element method (FEM), finite difference method (FDM), or peridynamics (PD) [82]. Regarding SA, the degradation mechanism comprises four interconnected modules: transport processes, chemical reactions, expansion forces, and mechanical responses [83]. Notable advancements include Jiang et al.’s [16] transport model integrating porosity and damage thresholds, Yu et al.’s [84] operator splitting approach for sequential computation of ion-reaction-mechanics processes, Gouder et al.’s [85] mixture theory-based sulfate simulation, Liu et al.’s [86] peridynamic analysis of expansion cracking, Zuo et al.’s [13] full-process chemo-mechanical model, and Li et al.’s [17] COMSOL-GEMS-MATLAB (a numerical simulation strategy that integrates the finite element software COMSOL Multiphysics with the geochemical modeling platform GEMS through MATLAB interface programming) coupled reaction-transport framework.

The interaction between freeze–thaw damage and sulfate erosion is influenced by temperature effects, changes in ion diffusion due to pore structure alterations, and chemical modifications in pore structure caused by variations in temperature and moisture transport [48]. The increase in porosity from FTCs reduces the expansion damage associated with the formation of calcite [87]. However, the combined effect of SA and FTCs, due to ongoing chemical damage or calcium dissolution, causes more damage to concrete than either effect alone. For instance, sulfate ion concentration in concrete during initial salt–freeze exposure is lower than in immersion environments due to pore changes; when concrete damage becomes severe, acid-soluble sulfate ion concentration exceeds immersion levels; additionally, sulfate reactivity in immersion environments surpasses that in freeze–thaw conditions due to temperature influences [39].

Some researchers have conducted modeling and simulation studies using traditional numerical simulation methods such as FEM [80] based on experimental results of macroscopic properties and microstructural changes under the combined effects of SA and FTCs. Zhu et al. [72] investigated the macroscopic properties and microstructural evolution of basalt fiber-reinforced concrete (BFRC) with varying dosages (0%, 0.1%, 0.2%, 0.3%) under combined Na_2_SO_4_ erosion and FTCs. Using ABAQUS finite element software, they simulated freeze–thaw cycling tests in Na_2_SO_4_ solution by equivalently applying thermal loads to represent volumetric expansion during sulfate freeze–thaw exposure. This equivalent thermal load method, implemented through user-defined subroutines, enabled simulation of the degradation process in BFRC under Na_2_SO_4_ freeze–thaw conditions, analyzing internal stress and strain field variations during testing. However, such approaches face limitations in mesh discretization and stress concentration issues.

**Figure 11:**
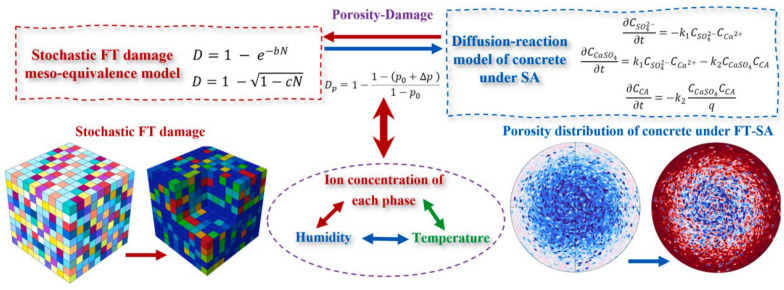
Degradation analysis method of concrete under combined sulfate attack and freeze–thaw cycles.

Current research on numerical simulation of concrete durability damage under combined SA and FTCs primarily focuses on multi-field coupling models. Liang et al. [87] developed a stochastic damage model proposing a mesoscale equivalent method for freeze–thaw damage that incorporates coupled environmental effects (temperature transitions, phase changes, and moisture transport). By integrating calcium leaching processes, diffusion-reaction modeling, and porosity variations, they established a mesoscale simulation approach for concrete under SA. Building upon this foundation, the team created a coupled mesoscale computational method—using porosity variation as an intermediate variable—to analyze damage evolution in concrete structures subjected to combined SA and FTC exposure, as shown in Figure 11.

**Figure 12:**
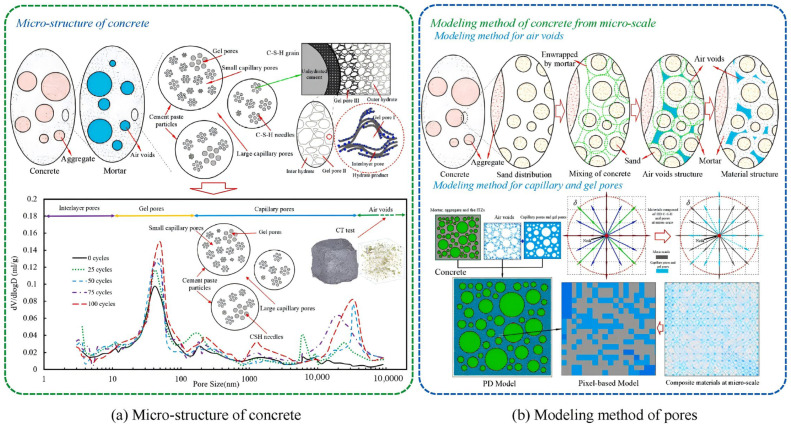
Modeling method of concrete on the micro-scale.

Peng et al. [88] conducted a study on the reinforced concrete prefabricated segments of bridge piers under seawater FTC conditions. By combining mercury intrusion porosimetry (MIP) and computed tomography (CT) to characterize concrete micropore structures with ultrasonic tomography for FTC damage distribution mapping, they developed a PeriDynamics (PD) numerical calculation method for salt–freeze damage prediction. This approach integrates the critical saturation theory, temperature distribution characteristics, pore crystallization theory, and pore pressure modeling. Using MATLAB-ABAQUS co-simulation, the PD-based computational framework (Figure 12) demonstrates exceptional capability in simulating pore accumulation/transformation and accurately characterizing concrete pore evolution behavior, as validated by experimental and computational results.

Zheng et al. [89] further investigated the durability of self-compacting concrete incorporating recycled coarse aggregates (RCA-SCC) under combined sulfate attack (SA) and freeze–thaw cycles (FTCs). Utilizing the GM(1,1) model grounded in gray system theory, they evaluated RCA-SCC performance degradation under coupled sulfate freeze–thaw exposure. Comparative analysis of four GM(1,1) variants revealed that the Even Gray Model (EGM) demonstrates superior predictive capability for estimating RCA-SCC service life under such synergistic degradation mechanisms.

Critically, while qualitative conclusions regarding concrete damage under combined SA-FTC exposure have emerged, no consensus exists on a unified theoretical framework describing cross-scale evolution—from microscale pore deterioration propagating to aggregate/cement paste/interfacial transition zone (ITZ) failure, and, ultimately, manifesting as macroscale concrete degradation. This knowledge gap stems from the inherent complexity of coupled chemo-physical damage processes spanning multiple spatial and temporal scales.

## 5. Conclusions

Based on recent research advancements, particularly systematic reviews of experimental studies, this paper synthesizes damage mechanisms, constitutive models, and numerical simulation methods for concrete subjected to combined sulfate attack (SA) and freeze–thaw cycles (FTCs). Key conclusions are summarized as follows:

(1) Complexity and Stage-Specificity of Damage Mechanisms: Compared to isolated SA or FTC exposure, durability deterioration under coupled SA-FTC involves synergistic interactions, including fatigue stress from FTCs, physical salt crystallization, and chemical expansion from sulfate–cement reactions. Key interactive effects—such as sulfate-induced freezing-point depression, increased initial saturation, and temperature-dependent sulfate transport—lead to a highly complex physicochemical deterioration process. Critically, these mechanisms exhibit time-variant synergistic/antagonistic interactions during damage evolution. Current research widely acknowledges that the deterioration process exhibits distinct stage-specific characteristics (e.g., three- or four-stage models). A consensus on concrete degradation mechanisms under combined SA-FTC exposure remains elusive. A unified and quantitative understanding remains lacking regarding transition thresholds between stages, the dominant versus secondary roles of various factors (such as salt type, concentration, and thermal history) in specific stages, and their interactive mechanisms (whether synergistic or antagonistic).
(2) Experimental Research and Limitations: Studies examining factors (e.g., solution concentration/type, test protocols, w/c ratio, air entrainment, fly ash, fiber reinforcement) have established standardized methodologies for quantifying SA-FTC damage via deterioration indicators (mass loss, relative dynamic modulus, compressive strength). However, current experimentation remains confined to material-level properties that rely on simplified laboratory conditions (e.g., water freezing–thawing under constant salt concentration), which diverge significantly from real-world exposure involving air freezing–water thawing, wet–dry cycles, and varying concentration–temperature histories, thereby constraining the extrapolation of laboratory findings to engineering practice. Moreover, studies remain largely focused on the material level, with a lack of systematic linkage to component- and structural-level performance (e.g., load-bearing capacity and stiffness degradation). Furthermore, systematic investigations into the effects of key environmental variables (e.g., cooling rate, minimum temperature and duration, humidity) and compound salt erosion mechanisms remain insufficient, hindering a comprehensive understanding and accurate prediction of durability in realistic service environments.
(3) Damage Modeling and Challenges: To quantify SA-FTC coupled damage, researchers have developed empirical and probabilistic models. Macroscale damage models derived from SA-FTC experimental data—typically formulated using exponential or Weibull functions—inadequately characterize concrete frost damage due to limitations in test duration and indicator selection, which curtails their generalizability and mechanistic insight. For models employing damage accumulation or probability distribution theories (e.g., Wiener or Weibull distributions), the definition and selection of damage variables critically govern model reliability. Moreover, both model types fail to explicitly capture the intrinsic mechanisms through which multifactorial influences govern concrete frost resistance, ultimately hindering mechanistic interpretation and broader applicability.
(4) Numerical Simulation and Current Bottlenecks: Both traditional (e.g., finite element method) and novel peridynamics (PD)-based methods have been applied to simulate concrete behavior under combined SA-FTCs. Nonetheless, research achievements remain sparse and constrained by reliance on limited experimental datasets for validation and parameterization. A major bottleneck is the absence of a predictive, multiscale simulation framework capable of integrating microstructural evolution with macroscopic performance under these complex environmental conditions. Critically, no multiscale numerical framework spanning micro-to-macro levels has been established to fully elucidate the durability damage mechanisms of SA-FTC. Consequently, developing a comprehensive theoretical damage model that integrates multifactorial mechanistic actions, supported by robust numerical simulations of durability degradation, represents a crucial direction for future research.
(5) Perspectives for Future Research: Future research must develop advanced testing protocols replicating real-field conditions, such as varying salt concentrations, thermal cycles, and wet–dry actions. Research progress hinges on advanced damage identification techniques, including post-exposure assessment methods and quantitative damage metrics. Cross-scale studies enabling multiscale characterization of concrete damage require deeper exploration. Such advancements are essential to clarify internal damage propagation processes, thereby providing theoretical and computational foundations for establishing multiscale damage models. High-fidelity multiscale numerical simulations, integrating chemo-thermo-mechanical coupling and phase-field modeling, should be established to bridge physicochemical processes with structural performance for predictive durability analysis.

## Figures and Tables

**Table 1:** Freezing and thawing experiments of concrete in various kinds and concentrations of salt solutions.

Salt Solutions	Specimen Dimension, Age	Scholar	w/b Ratio	Evaluation Indicators	Freeze–Thaw Method	Testing Intervals	Key Results
5% Na_2_SO_4_ (mass fraction)	40 mm × 40 mm × 160 mm, 28 d	Mu et al. [55]	0.44, 0.32, 0.26	Mass loss, RDEM	Rapid freeze–thaw (water freezing and water melting)	Every 20 cycles (≤300 cycles)	During the initial freeze–thaw phase, sulfate-induced damage is less severe compared to pure water exposure; however, in later stages, concrete with low water–binder ratio exhibits the most significant deterioration under sulfate freeze–thaw conditions.
3% NaCl and 5% Na_2_SO_4_ (mass fraction)	100 mm × l00 mm × l00 mm, 28 d	Zheng et al. [57]	0.44	Compressive strength, SEM, XRD	Rapid freeze–thaw (air freezing and air melting, −15 °C~8 °C), 12 h solution immersion every 10 cycles	Every 100 cycles (≤400 cycles)	Mechanical properties of concrete materials experience accelerated deterioration.
	100 mm × l00 mm × l00 mm and 100 mm × l00 mm × 300 mm, 28 d	Li et al. [58]	0.4	Mass loss, RDEM, compressive strength, elastic modulus	Slow freeze–thaw (air freezing and water melting), 4 h freezing in air at −18 °C to −20 °C, 4 h thawing while fully immersed in the mixed erosion solution at 18–20 °C	Every 100 cycles (≤300 cycles)	With the increase in freeze–thaw cycles, increased erosion of concrete specimen surface, and the apparent quality and relative dynamic modulus did not change significantly when both compressive strength and elastic modulus decreased significantly. Compressive strength and elastic modulus decreased by 26% and 35.1% after 300 freeze–thaw cycles.
1% Na_2_SO_4_, 5% Na_2_SO_4_ or 5% MgSO_4_ (mass fraction)	100 mm × l00 mm × 400 mm, 28 d + 60 d natural curing	Yuan et al. [59]	0.45	Damage layer thickness	Rapid freeze–thaw (water freezing and water melting)	Start at 150 cycles, then every 50 cycles (≤400 cycles)	With the increase of solution concentration, the promoting effect of freeze–thaw damage for the concrete with sulfate solution change to an inhibitory effect.
	100 mm × 100 mm × 400 mm for relatively elastic modulus and damage layer thickness test; 100 mm × 100 mm × 100 mm for compressive strength and SEM analysis	Jiang et al. [19]	0.45	RDEM, compressive strength, damage layer thickness, SEM	Rapid freeze–thaw (water freezing and water melting)	Start at 100 cycles, then every 50 cycles (≤400 cycles)	Concrete degradation rate in 1% Na_2_SO_4_ solution is faster than that in 5% Na_2_SO_4_ solution and in water.
	12 prisms of 100 mm × 100 mm × 400 mm, 28 d	Yuan et al. [60]	0.45	Damage layer thickness	Rapid freeze–thaw (water freezing and water melting)	Start at 150 cycles, then every 50 cycles (≤350 cycles)	Sodium sulfate acts as a promoter at low concentrations and an inhibitor at high ones.
3% NaCl and 3% Na_2_SO_4_ (mass fraction)	100 mm × 100 mm × 100 mm, 28 d	Lu et al. [52]	0.34	Mass, ultrasonic sound velocity value, NMR, SEM, XRD	Air freeze–water thaw–air freeze–air thaw cycle, specifically: 6 h in freezing chamber at −20 °C, 6 h immersed in salt solution at 20 °C, 6 h in freezing chamber at −20 °C, 6 h in ambient air at 20 °C	Every 25 cycles (≤300 cycles)	Durability life prediction model for concrete was established based on the Birnbaum–Saunders distribution.
Brine from a salt lake	40 mm × 40 mm × 160 mm, 28 d	Yu et al. [40]	0.60	RDEM, speed of sound in ultrasonic waves, Mass loss, XRD	Rapid freeze–thaw (water freezing and water melting)	Every 25 cycles	Salt solution has a positive effect by lowering the freezing point, while its negative effect is accelerating salt crystallization.
5% Na_2_SO_4_, 3.5% NaCl and 5% Na_2_SO_4_ (mass fraction)	100 mm × 100 mm × 400 mm, 28 d	Jin et al. [61]	0.46, 0.32	Ultrasonic transit time, ${\mathrm{SO}}_{4}^{2-}$ mass fraction of water-soluble and acid-soluble components, pore structure evolution, thermogravimetric curve	Rapid freeze–thaw (water freezing and water melting)	0, 50, 150, 200 cycles	An increase to double the sulfate ion reactivity and a 2.2-times enlargement of capillary pores were observed in concrete under mixed solution frost, compared to exposure to a 5% Na_2_SO_4_ solution.
10% Na_2_SO_4_,15% Na_2_SO_4_	100 mm × 100 mm × 100 mm, 28 d	Tian et al. [62]	0.45	Mass, RDEM, uniaxial compressive strength, CT	Slow freeze–thaw (air freeze: −18~−20 °C/4 h; solution thaw: 18~20 °C/4 h)	0, 20, 40, 60, 80, 100 cycles	Sulfate and freeze–thaw interaction exhibits two-stage deterioration: initial mitigation followed by later acceleration.
5% Na_2_SO_4_	120 cubes of 100 mm × 100 mm × 100 mm and 36 prisms of 100 mm × 100 mm × 400 mm, 28 d	Chen et al. [63]	0.35	Mass loss, RDEM, uniaxial compressive strength	Sulfate dry–wet cycling → Freeze–thaw cycling. Dry–wet cycle (24 h): 16 h immersion in 5% Na_2_SO_4_ solution, 6 h drying at 80 °C, 2 h cooling; Freeze–thaw cycle (6 h): Freezing 4 h at −20 ± 2 °C, Thawing 2 h at 5 ± 2 °C Alternating protocol: 16 days = 1 combined cycle (dry–wet + freeze–thaw)	Every cycle (16 d), 80 days (5 cycles)	Compressive strength-based GM(1,1) modeling for service life prediction of concrete.
5% Na_2_SO_4_, 5% MgSO_4_, and 3.5% NaCl compound salt solution	Φ100 × 50 mm, 150 d outdoor exposure	Duan et al. [64]	0.32 (fiber content is 0.9, 1.2, and 1.4 kg/m^3^)	Ultrasonic velocity, splitting tensile strength, SEM	Rapid freeze–thaw (water freezing and water melting), 2.5 h freeze/1.5 h thaw	Every 25 cycles (≤200 cycles)	Concrete damage degradation model with ultrasonic pulse velocity damage quantity as independent variable and relative splitting tensile strength as dependent variable.
5% Na_2_SO_4_, 10% Na_2_SO_4_	100 mm × 100 mm × 100 mm, 28 d	Xie et al. [65]	0.4 (fine aggregate selected 20% of the wind-sediment sand to replace the river sand)	Mass loss, compressive strength, SEM	Rapid freeze–thaw (−20 °C~20 °C, 4 h cycle)	0, 20, 40, 60, 80 cycles	Weibull function-based constitutive model for concrete damage using micro-damage variables.
3%, 6%, 9% Na_2_SO_4_	288 cube of 100 mm × 100 mm × 100 mm, 28 d	Gan et al. [24,45]	0.4	Mass change, compressive strength, splitting tensile strength, RDEM, CT	Salt immersion (15 d) + freeze–thaw cycling (25 cycles)	Every 25 cycles (≤200 cycles)	Expression for damage evolution of concrete under cyclic salt–freeze–thaw action with number of freeze–thaw cycles as independent variable.
20% Na_2_SO_4_	Φ50 × 100 mm, 28 d + 150 d immersion + 3 d drying	Xue et al. [48]	0.57	NMR, compressive permeability	Cyclic: 10 h 20% Na_2_SO_4_ solution immersion/8 h freeze (−20 °C)/6 h dry (65 °C)	0, 5, 10, 15, 20 cycles	Proportion of mesopores and macropores in concrete increases with the number of salt–freeze–thaw cycles.
10% Na_2_SO_4_	40 mm × 40 mm × 40 mm (compression) 40 mm × 40 mm × 160 mm (flexure)	Wang et al. [66]	0.18, 0.20, 0.22 (different amounts of silica powder and fiber)	Compressive/flexural strength, mass change, RDEM, SEM, XRD, TG-DTG, MIP	Multi-stage: 3 d high-temp + 30 d dry–wet + 2 d freeze–thaw + 2 d sulfate + 3 d low-temp (40 d/cycle ≈ 5 years)	Every cycle (≤6 cycles)	Wiener stochastic process-based life expectancy model using compressive strength as the state variable.
5% Na_2_SO_4_, mass fraction	40 mm × 40 mm × 160 mm	Liu et al. [21]	0.38	RDEM	Rapid freeze–thaw	Every 20 cycles (≤200 cycles)	Expression for freeze–thaw damage in concrete with dynamic elastic modulus as the variable.
6% Na_2_SO_4_, mass fraction	100 mm × 100 mm × 100 mm, 7 d	Zhang et al. [67]	0.26, 0.32, 0.38	Compressive strength	Rapid freeze–thaw (−23.3 °C~43.1 °C, 6 h freeze/8 h thaw, 24 h/cycle ≈ 1 year)	Every 10 cycles (≤100 cycles)	Variable-weighted buffer gm(1,1) model for early-age concrete strength using anti-corrosion coefficient as the parameter.
6% Na_2_SO_4_, mass fraction	100 mm × 100 mm × 100 mm, 7 d	Zhang et al. [68]	0.32 (inhibitor content of 0.00%, 0.05%, 0.10%, and 0.15%)	Appearance, mass loss, compressive strength, dynamic modulus	Rapid freeze–thaw (−23.3 °C~43.1 °C, 6 h freeze/8 h thaw, 24 h/cycle ≈ 1 year)	Every 10 cycles (≤100 cycles)	GM-GA-BP Model for early-age concrete service life prediction using number of freeze–thaw cycles as the parameter.
5% Na_2_SO_4_, 10% Na_2_SO_4_, mass fraction	100 mm × 100 mm × 100 mm 100 mm × 100 mm × 400 mm (RDME)	Dong et al. [42,69]	0.54	Compressive strength, dynamic elastic modulus, Mass, SEM, XRD, NMR	Rapid freeze–thaw	Every 25 cycles	Weibull stochastic probability distribution model for concrete service life prediction using compressive strength as the degradation index.
5% Na_2_SO_4_, mass fraction	100 mm × 100 mm × 100 mm 100 mm × 100 mm × 400 mm (RDME), 28 d	Xiao et al. [46]	0.45 (0%, 30%, 50%, 100% replacement rate of coarse recycled concrete aggregate)	Mass, RDEM, compressive strength	ASTM C666 (8 ± 2 °C to 17 ± 2 °C)	Every 25 cycles	Freeze–thaw random damage model for recycled concrete using a two-parameter Weibull probability distribution.
5% Na_2_SO_4_, mass fraction	100 mm × 100 mm × 100 mm 100 mm × 100 mm × 400 mm (RDME), 28 d	Wei et al. [30]	0.38 (0%, 50%, 100% replacement rate of coarse recycled concrete aggregate)	Mass, dynamic elastic modulus, NMR, Vickers hardness of ITZs	The specimen center’s temperature range during the freeze–thaw cycle test is − 18 °C to 5 °C	Every 25 cycles (≤300 cycles)	Pore structure damage model for concrete with comprehensive porosity parameters as variables.
0.89%, 3.7%, 7.4% Na_2_SO_4_, mass fraction	100 mm × 100 mm × 100 mm, 28 d	Zhu et al. [70]	0.38 (manufactured sand)	Sulfate content	Rapid freeze–thaw (−20 ± 2 °C to 8 ± 2 °C, 3–5 h/cycle)	Every 25 cycles (≤150 cycles)	Diffusion equation for concrete under sulfate freeze–thaw cycles with sulfate ion content as the independent variable.
5% Na_2_SO_4_ and 5% MgSO_4_, mass fraction	100 mm × 100 mm × 100 mm, cured in water for 28 d after demolding, and then cured for 23 months +2 d under laboratory conditions. Total curing time is 2 years.	Tanyildizi [71]	0.47	Mechanical properties, Mass, RDEM, SEM, EDS	Slow freeze–thaw (7 h at −20 ± 2 °C and 5 h at 20 ± 2 °C)	After 56 cycles	Samples exposed to sodium sulfate and freeze–thaw were less affected by the increase in cement dosage.
23 g/L Na_2_SO_4_, mass fraction	100 mm × 100 mm × 100 mm	Zhu et al. [72]	0.38 (0.1%, 0.2%, and 0.3% bulk accumulative amount of basalt fiber)	RDEM, mass loss, compressive/splitting strength	Rapid freeze–thaw (−20 ± 2 °C/6 h + 20 ± 2 °C/2 h)	Every 15 cycles (≤200 cycles)	Durability of concrete is improved by incorporating an appropriate amount of BF into the concrete to reduce the initial defects and slow down the rate of corrosive ions into the interior of the concrete.

Notes: The “rapid freezing method” refers to the procedure specified in either GB/T 50082 [73] or ASTM C666 [74]. However, in the test, the water in the specimen container is replaced with the corresponding salt solution. When the terms “solution-freezing/solution-thawing” or “air-freezing/solution-thawing” are used, “water”, here, specifically refers to the “salt solution”. Unless otherwise specified, the freezing–thawing temperatures are set as follows: at the end of both the freezing and thawing phases, the temperature at the center of the specimen shall be controlled at (−17 ± 2) °C and (+8 ± 2) °C, respectively. The freezing–thawing cycle time is set as follows: each complete freezing–thawing cycle shall be between 2 and 4 h, and the time allocated for thawing shall be not less than 1/4 of the total duration of each cycle.

## Data Availability

No new data were created or analyzed in this study. Data sharing is not applicable to this article.
